# *Chenopodium ambrosioides* L. Improves Phagocytic Activity and Decreases Bacterial Growth and the Systemic Inflammatory Response in Sepsis Induced by Cecal Ligation and Puncture

**DOI:** 10.3389/fmicb.2017.00148

**Published:** 2017-02-01

**Authors:** Carlos E. P. Rios, Afonso G. Abreu, Jose A. F. Braga Filho, Johnny R. Nascimento, Rosane N. M. Guerra, Flávia M. M. Amaral, Márcia C. G. Maciel, Flávia R. F. Nascimento

**Affiliations:** ^1^Laboratory of Immunophysiology, Federal University of MaranhãoSão Luís, Brazil; ^2^Programa de Pós-Graduação em Ciências da Saúde, Federal University of MaranhãoSão Luís, Brazil; ^3^Programa de Pós-Graduação em Biologia Parasitária, UniCEUMASão Luís, Brazil

**Keywords:** *Chenopodium ambrosioides*, sepsis, cecal ligation and puncture, phagocytes, cytokines

## Abstract

*Chenopodium ambrosioides* L. (Amaranthaceae) is often used in different kinds of vegetal preparations for medicinal purposes in many clinical situations. Some studies have demonstrated its anti-inflammatory and immunomodulatory properties. The aim of this work was to investigate the effect of prophylactic treatment with the hydroalcoholic crude extract (HCE) of *C. ambrosioides* and its hexanic fraction (HEX) on the control of bacterial growth, the activation of phagocytes and the control of the systemic inflammatory response in a sepsis experimental model. Animals were divided into three groups (*n* = 5/group): Control, which received only NaCl 0.9% solution; HCE, which received the crude extract; and HEX, which received the HEX of the extract. The animals received saline, HCE or HEX (5 mg/kg), subcutaneously (SC), 6 h before cecal ligation and puncture (CLP). Twelve hours after the CLP, the blood was collected to measure the serum cytokines and the animals were killed for the evaluation of colony-forming units (CFUs), cellular influx, and activation of phagocytes in the peritoneal cavity, measured by the secretion of hydrogen peroxide and nitric oxide production. The results showed that only HEX treatment inhibited bacterial growth in the peritoneum and inflammatory cellular influx, especially influx of macrophages and neutrophils. However, HCE and HEX treatments increased *ex vivo* hydrogen peroxide secretion and nitric oxide production by phagocytes and decreased the pro-inflammatory cytokines in the serum, indicating a systemic anti-inflammatory effect of both. In conclusion, *C. ambrosioides* treatment decreases bacterial growth likely by activation of phagocytes and, in parallel, ameliorates the general state of mice by reducing the systemic inflammatory response usually observed in sepsis.

## Introduction

*Chenopodium ambrosioides* L. (Amaranthaceae) is considered by the World Health Organization (WHO) as one of the species of plants frequently used for medicinal purposes in the world. In Brazil, it is widespread and has received several popular names, such as “Erva-de-Santa-Maria” and “Mastruz,” depending of the region. *C. ambrosioides* is frequently used as a diuretic and anti-inflammatory and healing agent (Kumar et al., 2007).

Some effects of *C. ambrosioides* have been confirmed, such as its anti-inflammatory (Trivellato-Grassi et al., 2013; Calado et al., 2015), anti-nociceptive (Ibironke and Ajiboye, 2007; Calado et al., 2015), antioxidant (Ibironke and Ajiboye, 2007; Patrício et al., 2008; Trivellato-Grassi et al., 2013) and anti-tumoural action (Nascimento et al., 2006) as well as its antimicrobial activity against *Trypanosoma cruzi* (Kiuchi et al., 2002), *Plasmodium falciparum* (Pollack et al., 1990; Cysne et al., 2016) and *Leishmania amazonensis* (Monzote et al., 2006; Patrício et al., 2008). Some studies have also demonstrated that *C. ambrosioides* has an immunostimulatory effect on lymphocytes (Rossi-Bergmann et al., 1997), and our group showed that this species induces activation of macrophages (Cruz et al., 2007).

Macrophages and neutrophils are crucial for controlling bacterial infections. These key cells migrate quickly to the infection site, recognize, phagocytose and destroy the microorganisms and release microbicidal agents, such as hydrogen peroxide and nitric oxide (Maciel et al., 2008). When activated, neutrophils synthesize several cytokines and chemokines responsible for recruiting and regulating the response of other cells at the lesion site. However, if the ability of the host is compromised or if the injury persists, the reaction that was initially contained becomes excessive and, sometimes, systemic, causing damage to the body (Benjamim et al., 2000).

Systemic inflammatory response syndrome (SIRS) can occur without the presence of a microorganism. When SIRS occurs in the presence of microorganisms, it is called sepsis, which is recognized by the involvement of the early activation of pro and anti-inflammatory responses together with modifications in non-immune pathways such as cardiovascular, neuronal, bioenergetic, metabolic, and coagulation pathways, all of which have prognostic significance. Septic shock is a subset of sepsis items in which circulatory, cellular and metabolic abnormalities are deep enough to increase mortality (Singer et al., 2016). SIRS has been recognized as a prominent factor in the mortality related to sepsis (Calandra and Cohen, 2005). The clinical manifestations of sepsis, such as fever, hyper coagulation and peripheral hypotension, are derived from the liberation of inflammatory mediators and cytokines such as IL-1β, IL-10, transforming growth factor β (TGF-β), tumor necrosis factor α (TNF-α) and macrophage chemotactic protein (MCP-1; Burkovskiy et al., 2013).

Most of the knowledge of the inflammatory mechanisms involved in sepsis was obtained using animal models (Buras et al., 2005). There are experimental models of sepsis, such as those resulting from the administration of viable microorganisms or microbial components and models of injury with consecutive release of intestinal microbiota [cecal ligation and puncture (CLP) or the introduction of a catheter into the ascending colon]. Among the experimental models of sepsis, CLP is the most widely used model because it more closely resembles the course of human sepsis (Baker et al., 1983; Benjamim, 2001).

Considering the potential of *C. ambrosioides* as an anti-inflammatory agent and a macrophage activator, the aim of this work was to investigate if the hydroalcoholic extract of *C. ambrosioides* and its HEX, used in a prophylactic way, could control the bacterial growth and systemic inflammatory reaction observed in sepsis induced by CLP.

## Materials and Methods

### Animals

Male Swiss mice, 8 to 12 weeks of age and weighing on average 25 g, were used (*n* = 5/group). The animals were obtained from the Central animal house of the Federal University of Maranhão (São Luís, Brazil), maintained at 26 ± 2°C, 44–56% relative humidity, under 12 h light-dark cycles and had free access to sterile food and acidified water. All procedures have been assessed and approved by the Committee of Ethics in Research at the Federal University of Maranhão (Protocol: 012975/2008-43).

### Plant Material

Leaves of *C. ambrosioides* were collected and identified at the Ático Seabra Herbarium (Federal University of Maranhão, São Luís, Brazil; voucher specimen N° 0998). They were dried at 37°C and later powdered. The dry powdered leaves (200 g) were then extracted with 1 L of ethanol (70%) and mixed each 8 h for 24 h. After this period, the hydroalcoholic extract was filtered, and then, the hydroalcoholic crude extract (HCE) was concentrated under low pressure. The yield obtained was 10.4% (w/w). Finally, the extract was dried, and the remainder was later lyophilized.

To obtain the HEX of *C. ambrosioides*, the lyophilized extract (21 g) was fractionated into a separating funnel with the addition of 400 mL of methanol and hexane until the hexane phase presented as colorless, resulting in the HEX. Subsequently, the HEX was concentrated, dried, and weighed. The yield of the HEX fraction was 1.3 g, and it was stored in sterile glass containers at 4°C.

### Experimental Design

The animals were initially divided into three groups: Control, which received a saline solution; HCE and HEX, which received treatment with HCE and HEX, respectively, at doses of 5 mg/kg. All treatments were administered through the subcutaneous route. After 6 h, the mice were submitted to the CLP as described below.

Polymicrobial sepsis was induced using the CLP method described by Benjamim et al. (2000), with modifications in the anesthetic used. Initially, the mice were anesthetized with 25 mg/kg ketamine hydrochloride and 20 mg/kg xylazine hydrochloride according to Machado et al. (2012). Then, a laparotomy was performed, and the cecum was mobilized, ligated below the cecal valve, and punctured 10 times with an 18-gauge needle to induce lethal sepsis. The cecum was placed back into the peritoneal cavity, and the abdomen was closed in two layers. Saline (0.5 mL/10 g body weight) was given subcutaneously to CLP animals for fluid resuscitation.

After 12 h of CLP, the blood of anesthetized mice was collected. The mice were euthanized with an overdose of anesthetic (150 mg/kg ketamine hydrochloride and 120 mg/kg xylazine hydrochloride).

### Peritoneal Cell Harvesting

Mouse peritoneal cells were harvested by washing the peritoneal cavity with 5 mL sterile ice-cold phosphate-buffered solution (PBS). Total cell numbers were estimated by counting cells with a haemocytometer. Differential cell counts were determined by cytospin preparations stained with Instant-Prov (Newprov, Pinhais, Brazil; Nascimento et al., 2015).

### Colony-Forming Unit (CFU) Determination

The skin of the abdomen was cut open in the midline after thorough disinfection and without injury to the muscle, and the peritoneal cavity was washed with 2 mL of sterile PBS. Aliquots of serial log dilutions of the peritoneal fluid obtained were plated on Mueller-Hinton agar dishes (Difco Laboratories, Detroit), and colony-forming units (CFUs) were counted after overnight incubation at 37°C; the results were expressed in the number of CFUs per peritoneal cavity.

### Determination of the Release of Hydrogen Peroxide (H_2_O_2_)

To evaluate H_2_O_2_ release, a horseradish peroxidase-dependent phenol red oxidation micro assay was used (Pick and Keisari, 1980; Pick and Mizel, 1981). In this assay, two million peritoneal cells were suspended in 1 mL freshly prepared phenol red solution that consisted of ice-cold Dulbecco’s PBS containing 5.5 mM dextrose, 0.56 mM phenol red (Sigma) and 8.5 U/mL horseradish peroxidase type II (Sigma). One hundred microliters of the cell suspension were added to each well and incubated in the presence or absence of 10 ng phorbol myristate acetate (PMA; Sigma) for 1 h at 37°C in a humid atmosphere containing 5% CO_2_ and 95% air. The plates were centrifuged once at 150 × *g* for 3 min, and the supernatants were collected and transferred to another plate. The reaction was stopped with 10 μL 1N NaOH. The absorbance was measured at 620 nm with a micro-plate reader (MR 5000, Dynatech Laboratories Inc., Gainesville, VA, USA). The conversion of the absorbance to μM H_2_O_2_ was done by comparison to a standard curve obtained with known concentrations of H_2_O_2_ (5–40 μM).

### Determination of Nitric Oxide Production

To measure NO, peritoneal cells were cultured in 100 μL of complete RPMI 1640 medium supplemented with 10 mM HEPES, 11 mM sodium bicarbonate, 100 U/mL penicillin, 100 μg/mL streptomycin, 2 mM L-glutamine, 23 mM L-asparagine, 1 mM folic acid, 0.1 mM pyruvic acid and 5% calf serum (FCS) for 48 h at 37°C in a humid atmosphere containing 5% CO_2_ and 95% air. After the incubation, 50 μL of supernatant was collected and incubated with an equal volume of Griess reagent (1% sulfanilamide/0.1% naphthalene diamine dihydrochloride/2.5% H_3_PO_4_) for 10 min at room temperature to quantify the accumulation of nitrite. The absorbance was determined at 550 nm. The conversion of the absorbance to μM of NO was done by comparison to a standard curve obtained with known concentrations (5–60 μM) of sodium nitrite diluted in RPMI medium (Ding et al., 1988).

### Counting of Spleen, Bone Marrow, and Lymph Node Cells

After the mice were sacrificed, the femur, spleen and inguinal lymph node were removed. The femur was perfused with 1 mL of PBS for the isolation of bone marrow cells. The spleen was removed, triturated with 5 mL PBS and passed through a silk sieve. Nine volumes of the cellular suspension were added to one volume of 0.05% crystal violet dissolved in 30% acetic acid, and the cells were counted using a bright-line Neubauer chamber (Sigma) under a common light microscopy at 400× magnification.

### Quantification of Cytokines

The cytometric bead array (CBA) technique was used for the quantification of TNF-α, MCP-1, IL-6, IL-10, IL-12 and IFN-γ in serum, as described by Maciel et al. (2014), using the Mouse inflammation cytokine kit (Becton Dickinson Biosciences, San Jose, CA, EUA).

### Statistical Analysis

The results are expressed as the mean ± standard deviation (SD) from five animals per group. Statistical evaluation was done by an ANOVA test followed by Neuman–Keuls *post hoc* test. Differences were considered significant at *P* < 0.05. The results were representative of two experiments.

## Results

### Prophylactic Treatment with HEX Decreases the Number of CFUs in the Peritoneal Cavity Fluid

**FIGURE 1:**
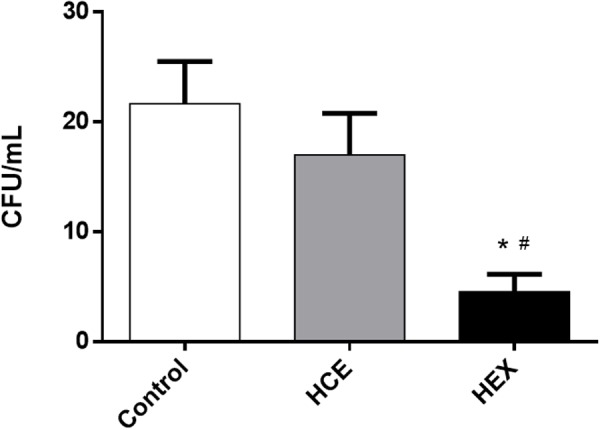
**Effect of hydroalcoholic crude extract (HCE) and hexanic fraction (HEX) of *C. ambrosioides* on the number of colony-forming units (CFUs) in the peritoneal cavity fluid of animals subjected to cecal ligation and puncture (CLP).** The number of CFUs in the peritoneal cavity was obtained 12 h after the CLP. The data were expressed as the mean ± SD (five animals/group). ^∗^*P* < 0.05 compared to the Control group; ^#^*P* < 0.05 compared to HCE.

Initially, considering the initial focus of the infection, the number of CFUs in the peritoneal cavity fluid was determined. The data showed that HEX prophylactic treatment reduced the number of CFUs when compared to that of the Control group. HCE prophylactic treatment did not alter the CFU number when compared to that of the Control group (**Figure 1**).

### Prophylactic Treatment with HCE and HEX Alters Inflammatory Cell Recruitment into the Peritoneal Cavity Induced by CLP

**FIGURE 2:**
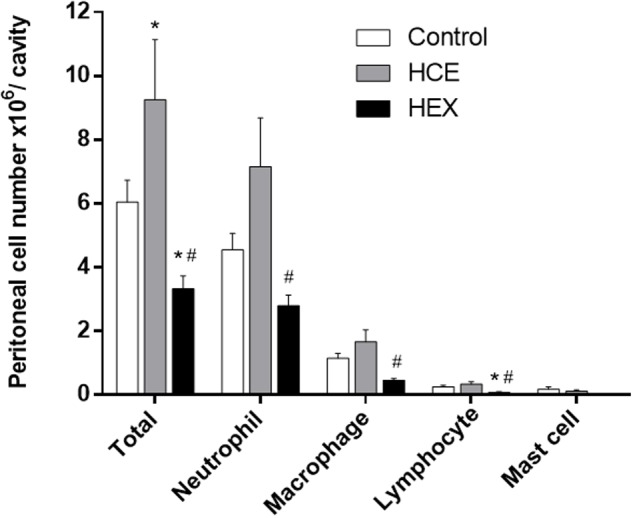
**Effect of prophylactic treatment with HCE and HEX of *C. ambrosioides* on inflammatory cell recruitment to the peritoneal cavity.** Total cell counts in the peritoneal cavity were obtained 12 h after the CLP. The results were expressed as the mean ± SD (five animals/group). ^∗^*P* < 0.05 compared to the Control group; ^#^*P* < 0.05 compared to HCE.

Considering the role of macrophages and neutrophils in the control of bacterial growth, we quantified the total and differential cell count in the peritoneal cavity. CLP was observed to induce an evident influx of inflammatory cells, with the majority being neutrophils. HCE treatment increased the influx of total cells, while treatment with HEX decreased this influx. The pattern of response in relation to macrophages and neutrophils was the same as that observed in the total cell count, although not significant. Treatment with HEX reduced the lymphocyte counts. The number of mast cells was similar in all three groups (**Figure 2**).

### Prophylactic Treatment with HCE and HEX Does Not Alter the Spleen and Bone Marrow Cell Count but Decreases the Lymph Node Cell Number

To verify if the differences in the peritoneal cell count could be related to alterations in the lymphoid organs, we quantified the cells in the bone marrow, spleen, and lymph node. Treatment with HCE and HEX did not alter the spleen and bone marrow cell count. However, both treatments reduced the number of cells in the lymph node, and this effect was more evident in the HCE treatment group (**Table 1**).

**Table 1:** Effect of treatment with hydroalcoholic crude extract (HCE) and hexanic fraction (HEX) of *C. ambrosioides* on bone marrow, spleen and lymph node cell counts in animals subjected to cecal ligation and puncture (CLP).

	Control	HCE	HEX
Bone marrow	4.63 ± 1.41	5.33 ± 2.21	3.70 ± 0.99
Spleen	3.64 ± 0.94	3.15 ± 0.72	2.58 ± 0.97
Lymph node	3.79 ± 1.08^a^	0.47 ± 0.17^∗^	1.09 ± 0.51^∗^

*^a^The results were expressed as the mean ± SD (five animals/group). ^∗^*P* < 0.05 compared to the Control group.*

### Prophylactic Treatment with HCE and HEX Induces an Increase in Hydrogen Peroxide (H_2_O_2_) Secretion and Nitric Oxide (NO) Production by Phagocytes

Considering the different effects observed with HCE and HEX treatment in relation to cellular influx to the infectious site and the control of bacterial growth, we analyzed the activation of phagocytes using their ability to secrete hydrogen peroxide and produce nitric oxide, two important microbicidal metabolites.

**FIGURE 3:**
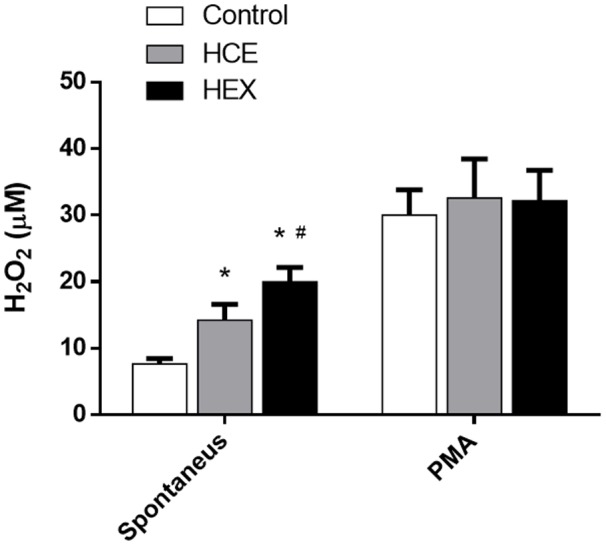
**Effect of HCE and HEX on the production of hydrogen peroxide in animals subjected to CLP.** The measurement of hydrogen peroxide in the peritoneal cavity was performed 12 h after the CLP. The data were expressed as the mean ± SD (five animals/group). ^∗^*P* < 0.05 compared to the Control group; ^#^*P* < 0.05 compared to HCE.

The data showed that HCE and HEX induced an increase in *ex vivo* spontaneous secretion of H_2_O_2._ The spontaneous H_2_O_2_ secretion was higher in the HEX group than in the HCE group. However, PMA increased the H_2_O_2_ release in all the groups with no difference between groups (**Figure 3**).

**FIGURE 4:**
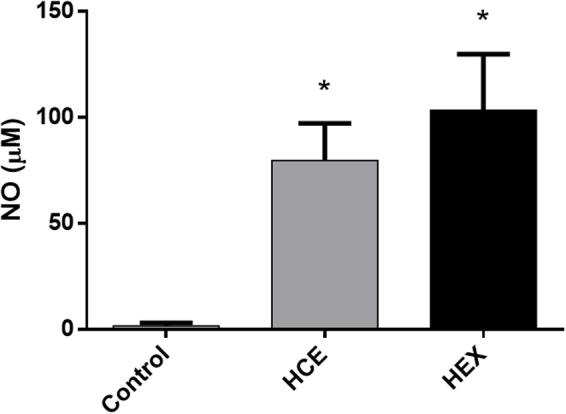
**Effect of HCE and HEX on the production of nitric oxide in animals subjected to CLP.** The measurement of nitric oxide in the peritoneal cavity was performed 12 h after the CLP. The data were expressed as the mean ± SD (five animals/group). ^∗^*P* < 0.05 compared to the Control group.

Hydroalcoholic crude extract and HEX treatment increased the *ex vivo* production of NO (**Figure 4**).

### Prophylactic Treatment with HCE and HEX Inhibits the Systemic Inflammatory Cytokines

**FIGURE 5:**
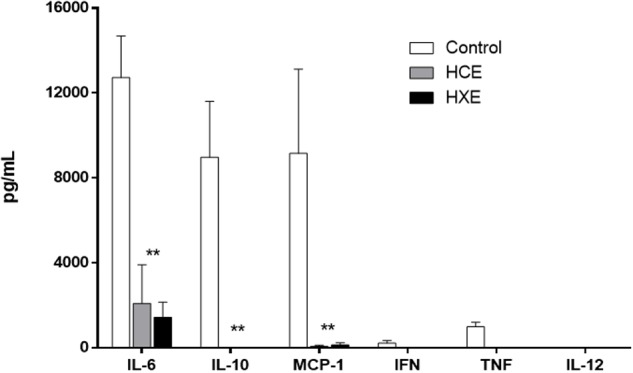
**Production of cytokines.** Quantification of TNF-α, MCP-1, IL-6, IL-10, IL-12 and IFN-γ in serum was performed 12 h after the CLP. The results were expressed as the mean ± SD (five animals/group). ^∗∗^*P* < 0.01 compared to HCE and HEX groups.

After the observation that treatment with HCE and HEX activates phagocytes at the site of infection and considering that the evolution of sepsis occurs in parallel to systemic inflammation, we chose to investigate some cytokines that are related to the progression of sepsis and the activation of phagocytes. Both HCE and HEX were observed to inhibit the production of the inflammatory cytokines IL-6, MCP-1, and TNF-α. The anti-inflammatory cytokine IL-10 was also decreased by both treatments. There was no effect of the treatments on IFN-γ levels. IL-12 was not detected in any of the groups (**Figure 5**).

## Discussion

Here, *C. ambrosioides* was demonstrated to induce a modulatory effect on the immune response with activation of phagocytes at the infection site but, at the same time, an anti-inflammatory profile, decreasing the inflammatory cytokines in the serum. However, this pattern of response was not necessarily related to the control of bacterial growth since only prophylactic treatment with HEX was able to reduce the bacterial counts.

Although the influx of inflammatory cells to the focus of the infection is crucial for the initial control of an infection, we observed here that the bacterial control does not necessarily seem to be related to the total number of cells in the peritoneal cavity since HCE and HEX induced divergent effects on inflammatory cell influx. The cellular recruitment is orchestrated by many components in the peritoneal cavity such as leukotriene, complement and IL-8, which are chemotactic to neutrophils and macrophages. Thus, it is reasonable to suppose that the reduction in cellular influx into the peritoneal cavity induced by HEX is related to a decrease in these chemotactic proteins, likely because of the smaller concentration of bacteria in this group.

These alterations in cell recruitment are not related to the toxicity of the treatments. Previous results obtained by our group showed that treatment with HCE at the dose used here has no toxicity (Pereira et al., 2010). In addition, when the cell count of the bone marrow and spleen was performed, there were no alterations observed, suggesting no immune toxicity. There was only a reduction in the number of lymph node cells, which is more related to a diminished proliferation due to the immunosuppression induced by *C. ambrosioides* than to toxicity. Interestingly, both treatments decreased the number of cells in the lymph node, despite a differential and opposing effect of the treatments on the influx of cells into the peritoneum.

Cellular recruitment to infectious sites naturally increases oxygen consumption by two factors: a higher number of neutrophils and increased cell activity. The increase in aerobic consumption is marked by the intensification of NADPH oxidase activity. This enzyme is involved in the production of reactive oxygen species, such hydrogen peroxide and anion superoxide, which are essential for the destruction of bacteria to avoid the progression and dissemination of infection to sepsis and septic shock (Pereira et al., 1998).

The spontaneous secretion of H_2_O_2_ was higher in the HCE and HEX groups than in the Control group. However, the treatments did not alter PMA-induced H_2_O_2_ secretion. PMA is a potent trigger of oxidative burst since it activates the kinase protein directly. However, action of PMA is evident only when the phagocytic cells are primed *in vivo*, as was the case for the cells tested here. Interestingly, HEX induced higher levels of H_2_O_2_ than HCE, which could explain the more intense microbicide response observed in HEX treatment. It is reasonable to suppose that there was no direct effect upon the peritoneal bacteria since the treatment was performed through a different route than that used to initiate the infection. The effects observed here are related more to the immunomodulatory action of *C. ambrosioides*.

In addition to H_2_O_2_, NO production was also increased by HCE and HEX. However, an exacerbated NO production is harmful to the body, leading to vasodilation and cytotoxicity (Cerqueira and Yoshida, 2002). This metabolite is a critical tool for the elimination of pathogens by the immune cells, limiting the spread of microorganisms and reducing the infection. In fact, *C. ambrosioides* directly induces macrophage activation as previously shown (Cruz et al., 2007).

Nevertheless, the quantification of CFUs in the peritoneal fluid shows that there was not a direct relation among the release of NO and H_2_O_2_ since HCE did not control bacterial growth, which was observed with treatment with HEX. However, this reduction seemed to direct the cellular influx since, in the HEX group, the diminished CFUs also inhibited the inflammatory cell influx. Thus, the organism was able to contain the infection without causing an exaggerated inflammatory response.

Finally, we investigated the effect of *C. ambrosioides* on the production of cytokines, which, among others mediators, are up-regulated and play important roles in the complex network of interactions associated with sepsis (Cavaillon et al., 2003). Specifically, we choose IFN-γ, TNF-a, IL-6, IL-10, IL-12, and MCP-1, which are involved in the recruitment and regulation of activation of macrophages and neutrophils to infectious site. In addition to their importance in this context, the exacerbated production of inflammatory cytokines can be crucial to a bad prognosis in sepsis.

We observed here that the systemic levels of cytokines were diminished in the groups that received HCE and HEX treatment when compared with that of the Control group. These results reflect the efficiency of prophylactic treatment with *C. ambrosioides* on reducing the systemic inflammation associated with sepsis. In an interesting way, HCE and HEX decreased the IL-10 cytokine, which is, in part, responsible for the decrease in inflammatory activity. It is possible that the anti-inflammatory mechanism involved in this case is related to the secondary metabolites, such as flavonoids, saponins and terpenoids present in this extract (Ibironke and Ajiboye, 2007; Castellanos, 2008; Patrício et al., 2008; Trivellato-Grassi et al., 2013). The production of inflammatory cytokines was controlled in the groups that received HCE and HEX treatment. These results reflect the efficiency of prophylactic treatment with *C. ambrosioides* on reducing systemic inflammation associated with sepsis.

The current treatment for sepsis is the administration of antibiotics and supportive care measures for the patient (Pammi and Weisman, 2015). We showed here that HCE and HEX treatment improved the local innate immunity, decreased bacterial growth and, concomitantly, avoided the systemic exacerbated inflammatory response usually observed in sepsis, thereby ameliorating the general state of mice and leading to a better prognosis.

## Ethics Statement

All performed procedures were authorized by the National Council for Animal Control and Experimentation (CONSEA) and approved by the Animal Use Ethics Committee of the UFMA under protocol number 23115.003819/2016-11.

## Author Contributions

Conceived and designed the experiments: CR, RG, MM, and FN. Performed the experiments: CR, JB, and JN. Analyzed the data: CR, AA, JB, JN, and FN. Contributed reagents/materials/analysis tools: FN and FA. Wrote the paper: CR, AA, MM, RG, FA, and FN.

## Conflict of Interest Statement

The authors declare that the research was conducted in the absence of any commercial or financial relationships that could be construed as a potential conflict of interest.
